# Supplementary figures and images for: Tissue-Specific Distribution of Eggs in The Definitive Host Drives Transcriptomic and Behavioral Differences in Schistosoma Mansoni Miracidia
Source: bioRxiv. 2025 Aug 19:2025.08.15.670345. Preprint. [Version 1] doi: 10.1101/2025.08.15.670345 (PMC12393456; doi:10.1101/2025.08.15.670345)

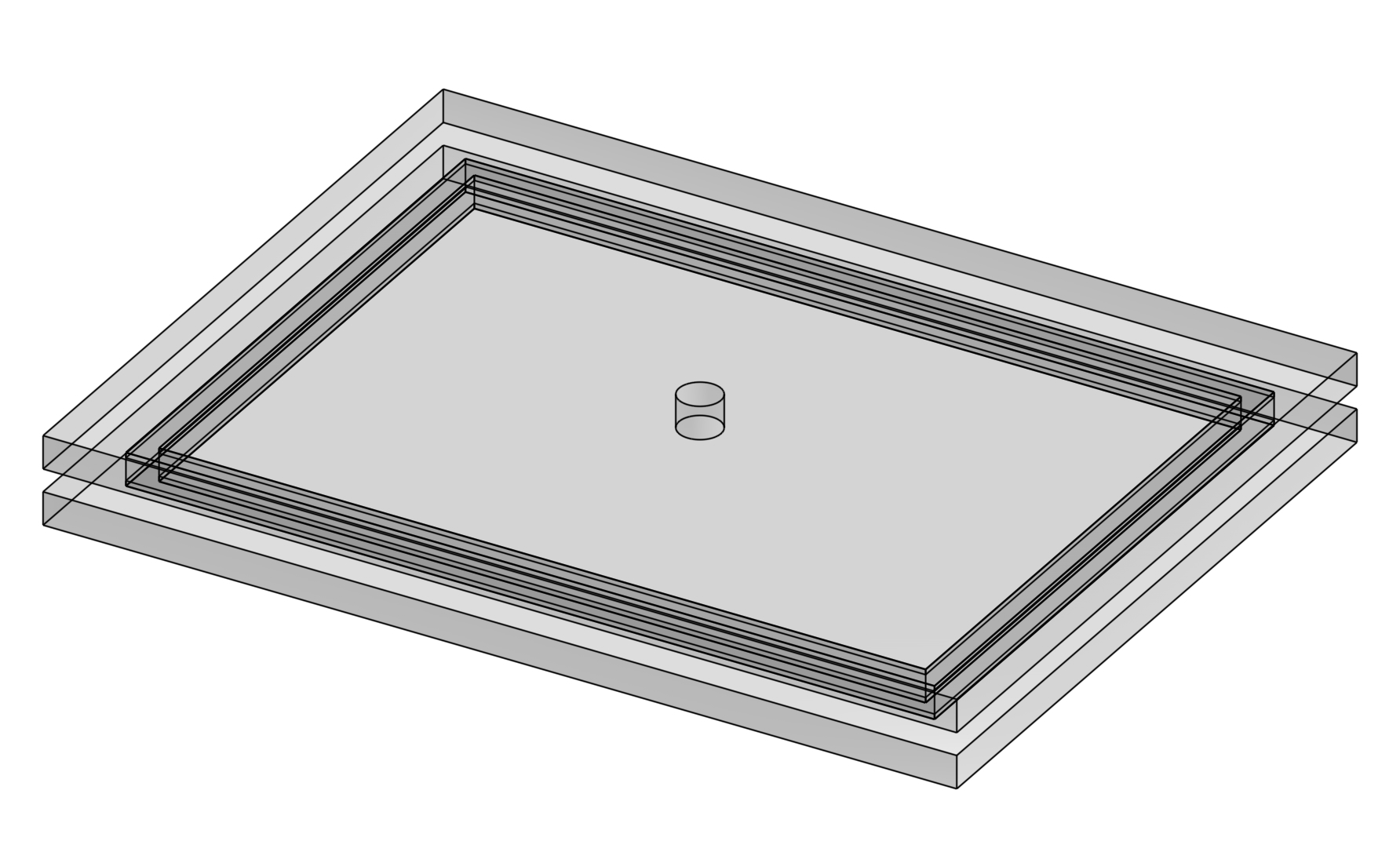

Supplement: Supplement 2 [file media-2.jpg]
